# 2,2′-(Quinoxaline-2,3-di­yl)diphenol dimethyl­formamide solvate

**DOI:** 10.1107/S1600536809054385

**Published:** 2010-01-16

**Authors:** Zhong-Lu You

**Affiliations:** a State Key Laboratory of Fine Chemicals, Dalian University of Technology, Dalian 116012, People’s Republic of China

## Abstract

In the title compound, C_20_H_14_N_2_O_2_·C_3_H_7_NO, the quinoxaline ring forms dihedral angles of 64.9 (2) and 30.9 (2)° with the two substituted benzene rings, which are themselves inclined at 58.4 (2)°. An intra­molecular O—H⋯N hydrogen bond occurs. In the crystal, mol­ecules are linked through inter­molecular O—H⋯O hydrogen bonds.

## Related literature

For details of cyanide-catalysed cyclizations *via* aldimine coupling, see: Reich *et al.* (2004 ▶).
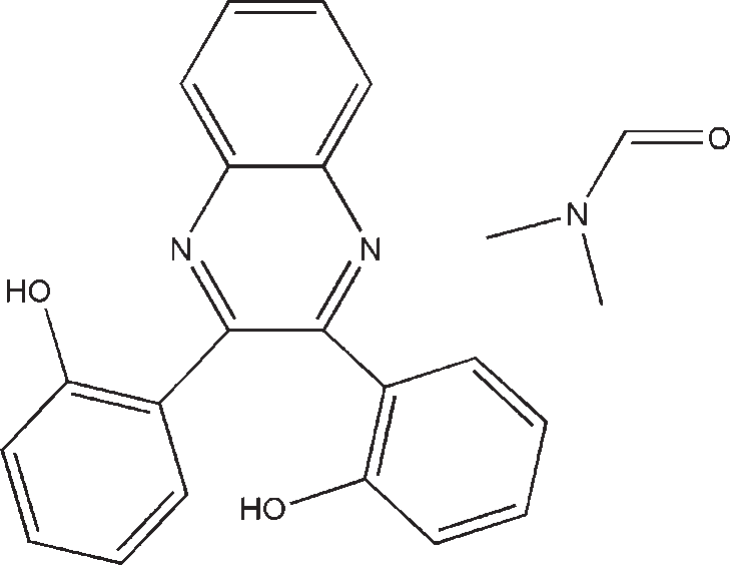


## Experimental

### 

#### Crystal data

C_20_H_14_N_2_O_2_·C_3_H_7_NO
*M* *_r_* = 387.43
Orthorhombic, 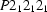

*a* = 9.759 (2) Å
*b* = 10.672 (2) Å
*c* = 19.049 (4) Å
*V* = 1983.9 (7) Å^3^
*Z* = 4
Mo *K*α radiation
μ = 0.09 mm^−1^
*T* = 298 K
0.23 × 0.21 × 0.20 mm

#### Data collection

Bruker SMART CCD area-detector diffractometer
Absorption correction: multi-scan (*SADABS*; Bruker, 2001 ▶) *T*_min_ = 0.980, *T*_max_ = 0.983
11593 measured reflections
4322 independent reflections
3434 reflections with *I* > 2σ(*I*)
*R*_int_ = 0.028

#### Refinement

*R*[*F* ^2^ > 2σ(*F* ^2^)] = 0.043
*wR*(*F* ^2^) = 0.102
*S* = 1.05
4322 reflections
266 parameters
H-atom parameters constrained
Δρ_max_ = 0.17 e Å^−3^
Δρ_min_ = −0.22 e Å^−3^

### 

Data collection: *SMART* (Bruker, 2007 ▶); cell refinement: *SAINT* (Bruker, 2007 ▶); data reduction: *SAINT*; program(s) used to solve structure: *SHELXTL* (Sheldrick, 2008 ▶); program(s) used to refine structure: *SHELXTL*; molecular graphics: *SHELXTL*; software used to prepare material for publication: *SHELXTL*.

## Supplementary Material

Crystal structure: contains datablocks global, I. DOI: 10.1107/S1600536809054385/sj2713sup1.cif

Structure factors: contains datablocks I. DOI: 10.1107/S1600536809054385/sj2713Isup2.hkl

Additional supplementary materials:  crystallographic information; 3D view; checkCIF report

## Figures and Tables

**Table 1:** Hydrogen-bond geometry (Å, °)

*D*—H⋯*A*	*D*—H	H⋯*A*	*D*⋯*A*	*D*—H⋯*A*
O2—H2⋯O3^i^	0.82	1.81	2.6172 (19)	168
O1—H1⋯N1	0.82	1.94	2.647 (2)	144

Symmetry code: (i) 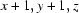.

**Fig. 1.**
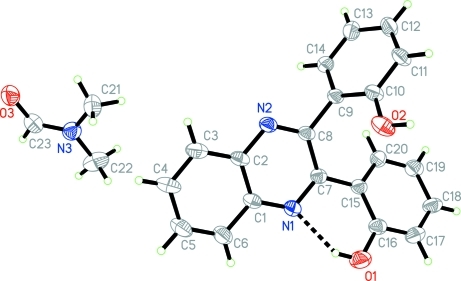
The molecular structure of the title compound with ellipsoids drawn at the 30% probability level. Hydrogen atoms are shown as spheres of arbitrary radius.

**Fig. 2.**
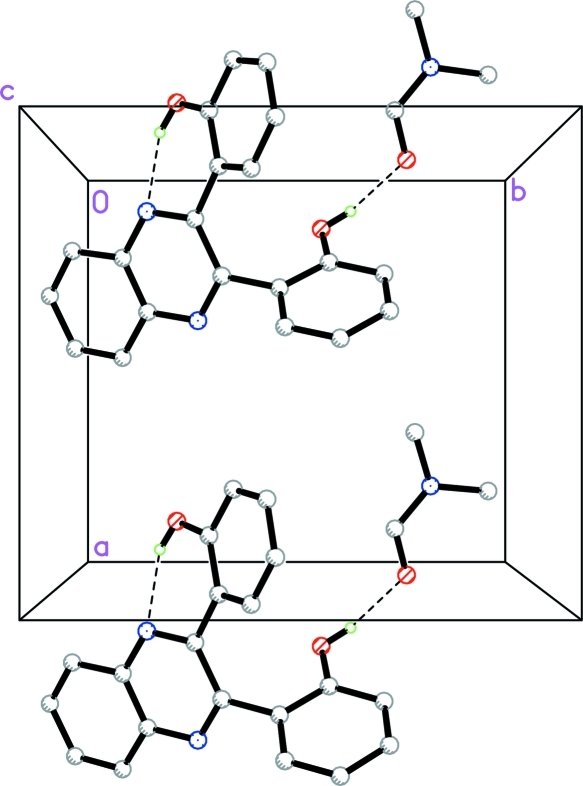
The molecular packing of the title compound, viewed along the c axis. Hydrogen bonds are drawn as dashed lines. Hydrogen atoms not involved in hydrogen bonding have been omitted.

## supplementary crystallographic information

### Comment

Reich and co-workers have reported a series of compounds with the cyanide-catalyzed cyclizations *via* aldimine coupling (Reich *et al.*, 2004), including the crystal structure of 2,2'-(quinoxaline-2,3-diyl)diphenol. In this paper, the title compound, Fig. 1, 2,2'-(quinoxaline-2,3-diyl)diphenol as the *N,N*dimethylformamide solvate is formed in an aldimine coupling reaction and its structure is reported here.

In the compound, the dimethylformamide molecule is linked to the 2,2'-(quinoxaline-2,3-diyl)diphenol molecule through an intermolecular O2—H2···O3 hydrogen bond (Table 1 and Fig. 2). The quinoxaline ring forms dihedral angles of 64.9 (2) and 30.9 (2)°, respectively, with the two substituted benzene rings C9-C14 and C15-C20. The dihedral angle between the two substituted benzene rings is 58.4 (2)°. An intramolecular O1—H1···N1 hydrogen bond (Table 1) is observed in the 2,2'-(quinoxaline-2,3-diyl)diphenol molecule.

### Experimental

The compound was prepared according to a literature procedure (Reich *et al.*, 2004). Yellow block-shaped crystals suitable for X-ray structural determination were obtained by slow diffusion of diethyl ether into a *N,N*dimethylformamide solution.

### Refinement

H atoms were placed in idealized positions and constrained to ride on their parent atoms, with C—H distances of 0.93-0.96 Å, O—H distances of 0.82 Å, and with *U*_iso_(H) = 1.2*U*_eq_(C) and 1.5*U*_eq_(C_methyl_ and O). In the absence of significant anomalous scattering effects, 1843 Friedel opposites were merged in the final refinement.

### Crystal data

**Table:**

C_20_H_14_N_2_O_2_·C_3_H_7_NO	*F*(000) = 816
*M**_r_* = 387.43	*D*_x_ = 1.297 Mg m^−^^3^
Orthorhombic, *P*2_1_2_1_2_1_	Mo *K*α radiation, λ = 0.71073 Å
Hall symbol: P 2ac 2ab	Cell parameters from 4283 reflections
*a* = 9.759 (2) Å	θ = 2.2–25.9°
*b* = 10.672 (2) Å	µ = 0.09 mm^−^^1^
*c* = 19.049 (4) Å	*T* = 298 K
*V* = 1983.9 (7) Å^3^	Block, yellow
*Z* = 4	0.23 × 0.21 × 0.20 mm

### Data collection

**Table:**

Bruker SMART CCD area-detector diffractometer	4322 independent reflections
Radiation source: fine-focus sealed tube	3434 reflections with *I* > 2σ(*I*)
graphite	*R*_int_ = 0.028
ω scans	θ_max_ = 27.0°, θ_min_ = 2.2°
Absorption correction: multi-scan (*SADABS*; Bruker, 2001)	*h* = −12→12
*T*_min_ = 0.980, *T*_max_ = 0.983	*k* = −13→10
11593 measured reflections	*l* = −24→19

### Refinement

**Table:**

Refinement on *F*^2^	Primary atom site location: structure-invariant direct methods
Least-squares matrix: full	Secondary atom site location: difference Fourier map
*R*[*F*^2^ > 2σ(*F*^2^)] = 0.043	Hydrogen site location: inferred from neighbouring sites
*wR*(*F*^2^) = 0.102	H-atom parameters constrained
*S* = 1.05	*w* = 1/[σ^2^(*F*_o_^2^) + (0.0529*P*)^2^ + 0.0554*P*] where *P* = (*F*_o_^2^ + 2*F*_c_^2^)/3
4322 reflections	(Δ/σ)_max_ = 0.001
266 parameters	Δρ_max_ = 0.17 e Å^−^^3^
0 restraints	Δρ_min_ = −0.22 e Å^−^^3^

### Special details

**Table:**

Geometry. All esds (except the esd in the dihedral angle between two l.s. planes) are estimated using the full covariance matrix. The cell esds are taken into account individually in the estimation of esds in distances, angles and torsion angles; correlations between esds in cell parameters are only used when they are defined by crystal symmetry. An approximate (isotropic) treatment of cell esds is used for estimating esds involving l.s. planes.
Refinement. Refinement of F^2^ against ALL reflections. The weighted R-factor wR and goodness of fit S are based on F^2^, conventional R-factors R are based on F, with F set to zero for negative F^2^. The threshold expression of F^2^ > 2sigma(F^2^) is used only for calculating R-factors(gt) etc. and is not relevant to the choice of reflections for refinement. R-factors based on F^2^ are statistically about twice as large as those based on F, and R- factors based on ALL data will be even larger.

### Fractional atomic coordinates and isotropic or equivalent isotropic displacement parameters (Å^2^)

**Table:**

	*x*	*y*	*z*	*U*_iso_*/*U*_eq_	
N1	0.87527 (15)	0.67113 (14)	0.14671 (8)	0.0496 (3)	
N2	0.61529 (14)	0.78228 (13)	0.14710 (8)	0.0494 (4)	
N3	0.22105 (17)	0.28969 (17)	0.15246 (8)	0.0639 (4)	
O1	1.13645 (14)	0.73589 (16)	0.15603 (8)	0.0771 (4)	
H1	1.0673	0.6979	0.1681	0.116*	
O2	0.83600 (16)	1.05141 (12)	0.16631 (7)	0.0695 (4)	
H2	0.8792	1.1162	0.1731	0.104*	
O3	0.00473 (16)	0.23971 (15)	0.18290 (9)	0.0795 (5)	
C1	0.76781 (19)	0.61476 (16)	0.18052 (9)	0.0483 (4)	
C2	0.63779 (19)	0.66962 (16)	0.18002 (9)	0.0486 (4)	
C3	0.5287 (2)	0.61110 (19)	0.21599 (11)	0.0639 (5)	
H3	0.4419	0.6472	0.2163	0.077*	
C4	0.5521 (3)	0.5016 (2)	0.25004 (11)	0.0711 (6)	
H4	0.4808	0.4635	0.2744	0.085*	
C5	0.6807 (2)	0.4452 (2)	0.24920 (11)	0.0721 (6)	
H5	0.6935	0.3691	0.2721	0.087*	
C6	0.7878 (2)	0.49954 (18)	0.21537 (11)	0.0641 (5)	
H6	0.8735	0.4613	0.2152	0.077*	
C7	0.85364 (16)	0.77757 (15)	0.11304 (8)	0.0430 (4)	
C8	0.71927 (17)	0.83557 (15)	0.11509 (8)	0.0433 (4)	
C9	0.68921 (17)	0.96032 (16)	0.08390 (9)	0.0464 (4)	
C10	0.75162 (19)	1.06703 (16)	0.11096 (9)	0.0528 (5)	
C11	0.7215 (2)	1.18364 (17)	0.08143 (11)	0.0638 (5)	
H11	0.7619	1.2557	0.0994	0.077*	
C12	0.6323 (2)	1.1920 (2)	0.02580 (12)	0.0684 (6)	
H12	0.6153	1.2697	0.0055	0.082*	
C13	0.5678 (2)	1.0874 (2)	−0.00042 (12)	0.0676 (5)	
H13	0.5062	1.0943	−0.0375	0.081*	
C14	0.59580 (18)	0.97191 (18)	0.02901 (11)	0.0550 (5)	
H14	0.5517	0.9009	0.0119	0.066*	
C15	0.97327 (16)	0.83049 (16)	0.07577 (9)	0.0453 (4)	
C16	1.10760 (18)	0.80861 (18)	0.09958 (9)	0.0529 (4)	
C17	1.2185 (2)	0.8622 (2)	0.06566 (11)	0.0625 (5)	
H17	1.3065	0.8496	0.0830	0.075*	
C18	1.1996 (2)	0.9338 (2)	0.00648 (10)	0.0633 (5)	
H18	1.2746	0.9701	−0.0157	0.076*	
C19	1.0700 (2)	0.95219 (19)	−0.02002 (10)	0.0615 (5)	
H19	1.0576	0.9991	−0.0607	0.074*	
C20	0.95835 (19)	0.90080 (18)	0.01386 (9)	0.0523 (4)	
H20	0.8712	0.9130	−0.0047	0.063*	
C21	0.2074 (3)	0.4182 (2)	0.17613 (15)	0.0940 (8)	
H21A	0.2527	0.4731	0.1436	0.141*	
H21B	0.2483	0.4268	0.2217	0.141*	
H21C	0.1120	0.4399	0.1787	0.141*	
C22	0.3494 (2)	0.2530 (3)	0.11988 (14)	0.0951 (8)	
H22A	0.3473	0.1649	0.1095	0.143*	
H22B	0.4238	0.2704	0.1514	0.143*	
H22C	0.3619	0.2993	0.0772	0.143*	
C23	0.1181 (2)	0.2123 (2)	0.15743 (10)	0.0660 (5)	
H23	0.1303	0.1311	0.1408	0.079*	

### Atomic displacement parameters (Å^2^)

**Table:**

	*U*^11^	*U*^22^	*U*^33^	*U*^12^	*U*^13^	*U*^23^
N1	0.0505 (8)	0.0416 (8)	0.0568 (8)	−0.0064 (7)	−0.0007 (7)	0.0007 (7)
N2	0.0512 (8)	0.0368 (8)	0.0601 (8)	−0.0058 (6)	0.0123 (7)	−0.0042 (6)
N3	0.0591 (10)	0.0707 (11)	0.0620 (9)	0.0009 (9)	0.0025 (8)	−0.0038 (8)
O1	0.0513 (8)	0.0915 (11)	0.0886 (10)	−0.0128 (8)	−0.0080 (7)	0.0264 (9)
O2	0.0955 (11)	0.0479 (8)	0.0652 (8)	−0.0194 (7)	−0.0054 (7)	−0.0031 (6)
O3	0.0707 (10)	0.0683 (10)	0.0996 (11)	−0.0145 (8)	0.0137 (9)	−0.0173 (8)
C1	0.0572 (10)	0.0390 (8)	0.0486 (9)	−0.0130 (8)	0.0007 (8)	−0.0009 (7)
C2	0.0576 (10)	0.0378 (9)	0.0504 (9)	−0.0130 (9)	0.0080 (8)	−0.0041 (7)
C3	0.0657 (12)	0.0562 (12)	0.0697 (12)	−0.0169 (10)	0.0190 (10)	0.0010 (9)
C4	0.0841 (16)	0.0620 (13)	0.0673 (13)	−0.0310 (12)	0.0107 (12)	0.0086 (10)
C5	0.0904 (17)	0.0550 (13)	0.0709 (12)	−0.0213 (12)	−0.0100 (12)	0.0199 (10)
C6	0.0700 (12)	0.0502 (11)	0.0721 (12)	−0.0085 (10)	−0.0075 (11)	0.0112 (10)
C7	0.0475 (9)	0.0362 (8)	0.0454 (8)	−0.0051 (7)	0.0043 (7)	−0.0075 (7)
C8	0.0478 (8)	0.0343 (8)	0.0478 (8)	−0.0065 (7)	0.0088 (8)	−0.0061 (7)
C9	0.0470 (9)	0.0364 (8)	0.0559 (9)	−0.0021 (7)	0.0157 (8)	−0.0025 (7)
C10	0.0645 (11)	0.0408 (9)	0.0530 (10)	−0.0061 (8)	0.0175 (9)	−0.0023 (8)
C11	0.0821 (13)	0.0348 (9)	0.0747 (13)	−0.0040 (10)	0.0253 (11)	−0.0012 (9)
C12	0.0776 (14)	0.0490 (12)	0.0786 (14)	0.0106 (11)	0.0244 (12)	0.0142 (10)
C13	0.0598 (12)	0.0668 (14)	0.0762 (13)	0.0110 (11)	0.0068 (10)	0.0094 (11)
C14	0.0470 (10)	0.0502 (11)	0.0679 (11)	0.0005 (8)	0.0102 (9)	−0.0015 (9)
C15	0.0478 (9)	0.0362 (9)	0.0519 (9)	−0.0041 (7)	0.0088 (8)	−0.0068 (7)
C16	0.0544 (10)	0.0481 (10)	0.0564 (10)	−0.0085 (8)	0.0057 (8)	−0.0063 (8)
C17	0.0482 (10)	0.0697 (13)	0.0696 (12)	−0.0101 (10)	0.0085 (10)	−0.0117 (10)
C18	0.0584 (12)	0.0635 (13)	0.0681 (12)	−0.0121 (10)	0.0250 (10)	−0.0117 (10)
C19	0.0715 (13)	0.0592 (12)	0.0539 (10)	−0.0016 (10)	0.0217 (9)	−0.0018 (9)
C20	0.0548 (10)	0.0509 (10)	0.0513 (10)	0.0036 (9)	0.0120 (8)	−0.0033 (8)
C21	0.0760 (15)	0.0876 (18)	0.119 (2)	−0.0221 (15)	0.0046 (15)	−0.0334 (16)
C22	0.0715 (15)	0.128 (2)	0.0853 (16)	0.0205 (16)	0.0133 (12)	0.0106 (16)
C23	0.0809 (15)	0.0573 (13)	0.0598 (12)	0.0019 (11)	0.0001 (11)	−0.0042 (9)

### Geometric parameters (Å, °)

**Table:**

N1—C7	1.321 (2)	C9—C14	1.393 (3)
N1—C1	1.370 (2)	C10—C11	1.397 (3)
N2—C8	1.313 (2)	C11—C12	1.374 (3)
N2—C2	1.374 (2)	C11—H11	0.9300
N3—C23	1.304 (3)	C12—C13	1.376 (3)
N3—C21	1.450 (3)	C12—H12	0.9300
N3—C22	1.452 (3)	C13—C14	1.381 (3)
O1—C16	1.356 (2)	C13—H13	0.9300
O1—H1	0.8200	C14—H14	0.9300
O2—C10	1.348 (2)	C15—C20	1.405 (2)
O2—H2	0.8200	C15—C16	1.407 (3)
O3—C23	1.243 (2)	C16—C17	1.384 (3)
C1—C2	1.398 (3)	C17—C18	1.375 (3)
C1—C6	1.411 (3)	C17—H17	0.9300
C2—C3	1.412 (2)	C18—C19	1.376 (3)
C3—C4	1.356 (3)	C18—H18	0.9300
C3—H3	0.9300	C19—C20	1.380 (3)
C4—C5	1.393 (3)	C19—H19	0.9300
C4—H4	0.9300	C20—H20	0.9300
C5—C6	1.358 (3)	C21—H21A	0.9600
C5—H5	0.9300	C21—H21B	0.9600
C6—H6	0.9300	C21—H21C	0.9600
C7—C8	1.451 (2)	C22—H22A	0.9600
C7—C15	1.479 (2)	C22—H22B	0.9600
C8—C9	1.487 (2)	C22—H22C	0.9600
C9—C10	1.391 (2)	C23—H23	0.9300
			
C7—N1—C1	118.91 (15)	C11—C12—H12	119.4
C8—N2—C2	117.87 (15)	C13—C12—H12	119.4
C23—N3—C21	120.37 (19)	C12—C13—C14	119.1 (2)
C23—N3—C22	121.7 (2)	C12—C13—H13	120.5
C21—N3—C22	117.9 (2)	C14—C13—H13	120.5
C16—O1—H1	109.5	C13—C14—C9	120.89 (19)
C10—O2—H2	109.5	C13—C14—H14	119.6
N1—C1—C2	120.52 (15)	C9—C14—H14	119.6
N1—C1—C6	119.86 (18)	C20—C15—C16	117.11 (15)
C2—C1—C6	119.61 (17)	C20—C15—C7	121.67 (15)
N2—C2—C1	120.99 (15)	C16—C15—C7	121.18 (15)
N2—C2—C3	119.22 (18)	O1—C16—C17	116.38 (17)
C1—C2—C3	119.74 (17)	O1—C16—C15	122.97 (16)
C4—C3—C2	119.1 (2)	C17—C16—C15	120.65 (17)
C4—C3—H3	120.4	C18—C17—C16	120.49 (19)
C2—C3—H3	120.4	C18—C17—H17	119.8
C3—C4—C5	121.28 (19)	C16—C17—H17	119.8
C3—C4—H4	119.4	C17—C18—C19	120.25 (18)
C5—C4—H4	119.4	C17—C18—H18	119.9
C6—C5—C4	121.0 (2)	C19—C18—H18	119.9
C6—C5—H5	119.5	C18—C19—C20	119.84 (19)
C4—C5—H5	119.5	C18—C19—H19	120.1
C5—C6—C1	119.3 (2)	C20—C19—H19	120.1
C5—C6—H6	120.4	C19—C20—C15	121.53 (18)
C1—C6—H6	120.4	C19—C20—H20	119.2
N1—C7—C8	119.88 (14)	C15—C20—H20	119.2
N1—C7—C15	115.80 (15)	N3—C21—H21A	109.5
C8—C7—C15	124.31 (15)	N3—C21—H21B	109.5
N2—C8—C7	121.74 (15)	H21A—C21—H21B	109.5
N2—C8—C9	114.88 (15)	N3—C21—H21C	109.5
C7—C8—C9	123.34 (14)	H21A—C21—H21C	109.5
C10—C9—C14	119.49 (17)	H21B—C21—H21C	109.5
C10—C9—C8	119.91 (16)	N3—C22—H22A	109.5
C14—C9—C8	120.57 (15)	N3—C22—H22B	109.5
O2—C10—C9	117.14 (15)	H22A—C22—H22B	109.5
O2—C10—C11	123.62 (17)	N3—C22—H22C	109.5
C9—C10—C11	119.22 (18)	H22A—C22—H22C	109.5
C12—C11—C10	120.10 (19)	H22B—C22—H22C	109.5
C12—C11—H11	120.0	O3—C23—N3	124.4 (2)
C10—C11—H11	120.0	O3—C23—H23	117.8
C11—C12—C13	121.16 (19)	N3—C23—H23	117.8

### Hydrogen-bond geometry (Å, °)

**Table:**

*D*—H···*A*	*D*—H	H···*A*	*D*···*A*	*D*—H···*A*
O2—H2···O3^i^	0.82	1.81	2.6172 (19)	168
O1—H1···N1	0.82	1.94	2.647 (2)	144

Symmetry codes: (i) *x*+1, *y*+1, *z*.
